# A199 MODELS OF OUTPATIENT INFLAMMATORY BOWEL DISEASE CARE DELIVERY: A SCOPING REVIEW

**DOI:** 10.1093/jcag/gwae059.199

**Published:** 2025-02-10

**Authors:** E Kuenzig, N Rohatinsky, T A Sheekha, J Im, M Huestis, C Seow, G G Kaplan, G Nguyen, E Benchimol

**Affiliations:** The Hospital for Sick Children, Toronto, ON, Canada; University of Saskatchewan, Saskatoon, SK, Canada; University of Saskatchewan, Saskatoon, SK, Canada; The Hospital for Sick Children, Toronto, ON, Canada; The Hospital for Sick Children, Toronto, ON, Canada; University of Calgary, Calgary, AB, Canada; University of Calgary, Calgary, AB, Canada; University of Toronto, Toronto, ON, Canada; The Hospital for Sick Children, Toronto, ON, Canada

## Abstract

**Background:**

The rapidly increasing prevalence of inflammatory bowel disease (IBD) in Canada, combined with an aging population at higher risk of age-related comorbidities, will place a significant strain on gastroenterology clinics and the broader healthcare system. Changes in care delivery are necessary to meet the growing demand for health services among people living with IBD.

**Aims:**

To understand the extent and type of evidence focused on models of outpatient care delivery for disease management in individuals with IBD.

**Methods:**

A scoping review was conducted by searching MEDLINE, EMBASE, CINAHL, and PsycINFO (from inception to March 4, 2024) to identify studies describing or evaluating models of care for managing people with IBD in outpatient settings. We included English language published studies of any type (primary studies, reviews, and opinion pieces) focusing on any age group, specific aspects of care (e.g., preconception, transition from pediatric to adult care), or setting (e.g., remote care or monitoring). Studies were screened for eligibility in duplicate, and conflicts were resolved by consensus. Heat maps were created to synthesize the evidence and summarize the literature recommendations.

**Results:**

Our search yielded 13,146 records, of which 209 met our inclusion criteria. These included 88 quantitative studies, 11 qualitative studies, 18 mixed methods studies, and 21 systematic or scoping reviews; the remaining studies consisted of clinical practice guidelines, narrative reviews, or perspectives. The models of care evaluated, along with their outcomes, are summarized in Figure A. Many interventions resulted in high levels of patient and clinician satisfaction, as well as reduced healthcare utilization and costs (direct, indirect, and out-of-pocket). However, data on other outcomes were mixed, with many studies being underpowered to detect significant improvements in patient outcomes. Gastroenterologists, nurses, mental health professionals (e.g., social workers, psychologists, psychiatrists), and dietitians were consistently identified as key members of multidisciplinary teams (Figure B).

**Conclusions:**

Several innovative outpatient models of IBD care have been proposed. The recommendations from these studies can guide adaptations in the Canadian healthcare system, addressing the growing healthcare needs of the increasing number of people living with IBD.

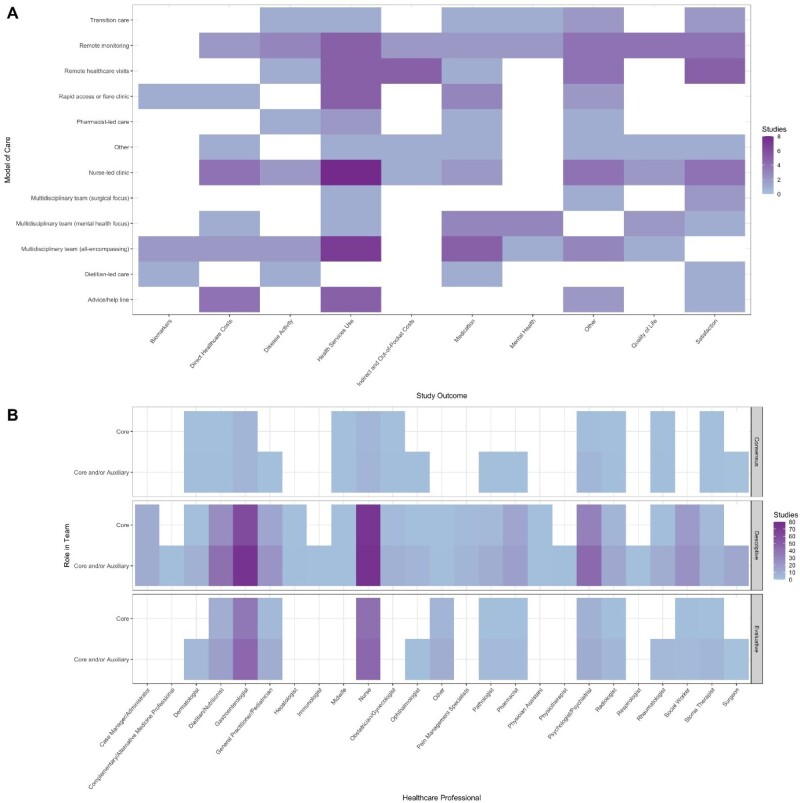

Heat maps describing (A) the evaluated models of outpatient inflammatory bowel disease care and their outcomes and (B) the recommended core and auxiliary members of multidisciplinary teams included in the described and evaluated models of care

**Funding Agencies:**

None

